# Barriers to, and Facilitators of, Checking Drugs for Adulterants in the Era of Fentanyl and Xylazine: Qualitative Study

**DOI:** 10.2196/56755

**Published:** 2024-07-03

**Authors:** Ian David Aronson, Mary-Andrée Ardouin-Guerrier, Juan Esteban Baus, Alex S Bennett

**Affiliations:** 1 School of Global Public Health New York University New York, NY United States; 2 Center for Technology-based Education and Community Health NDRI-USA New York, NY United States

**Keywords:** overdose, overdoses, fentanyl, xylazine, benzodiazepines, adulterants, drug, drugs, substance, substances, illicit drug, illicit drugs, drug test, drug testing, drug checking, qualitative, interview, interviews, digital health, digital technology, digital intervention, digital interventions, technological intervention, technological interventions, technology-based intervention, technology-based interventions

## Abstract

**Background:**

Overdose deaths continue to reach new records in New York City and nationwide, largely driven by adulterants such as fentanyl and xylazine in the illicit drug supply. Unknowingly consuming adulterated substances dramatically increases risks of overdose and other health problems, especially when individuals consume multiple adulterants and are exposed to a combination of drugs they did not intend to take. Although test strips and more sophisticated devices enable people to check drugs for adulterants including fentanyl and xylazine prior to consumption and are often available free of charge, many people who use drugs decline to use them.

**Objective:**

We sought to better understand why people in the New York City area do or do not check drugs before use. We plan to use study findings to inform the development of technology-based interventions to encourage consistent drug checking.

**Methods:**

In summer 2023, team members who have experience working with people who use drugs conducted 22 semistructured qualitative interviews with a convenience sample of people who reported illicit drug use within the past 90 days. An interview guide examined participants’ knowledge of and experience with adulterants including fentanyl, xylazine, and benzodiazepines; using drug testing strips; and whether they had ever received harm reduction services. All interviews were audio recorded, transcribed, and analyzed for emerging themes.

**Results:**

Most participants lacked knowledge of adulterants, and only a few reported regularly checking drugs. Reasons for not checking included lacking convenient access to test supplies, or a place to check samples out of the public’s view, as well as time considerations. Some participants also reported a strong belief that they were not at risk from fentanyl, xylazine, or other adulterants because they exclusively used cocaine or crack, or that they were confident the people they bought drugs from would not sell them adulterated substances. Those who did report testing their drugs described positive interactions with harm reduction agency staff.

**Conclusions:**

New forms of outreach are needed not only to increase people’s knowledge of adulterated substances and awareness of the increasing risks they pose but also to encourage people who use drugs to regularly check their substances prior to use. This includes new intervention messages that highlight the importance of drug checking in the context of a rapidly changing and volatile drug supply. This messaging can potentially help normalize drug checking as an easily enacted behavior that benefits public health. To increase effectiveness, messages can be developed with, and outreach can be conducted by, trusted community members including people who use drugs and, potentially, people who sell drugs. Pairing this messaging with access to no-cost drug-checking supplies and equipment may help address the ongoing spiral of increased overdose deaths nationwide.

## Introduction

The United States has set new annual records for overdose deaths almost every year for more than 20 years [1]. Much of this increase is due to a rapidly changing and unpredictable drug supply that can pose myriad health risks [2]. Nationally, more than 107,000 people died from overdose in the 12-month period ending in March 2022 [3], and more people in New York City died from opioid overdose than ever before [4,5]. Disparities in overdose mortality based on race and ethnicity are now being observed, with a tripling of overdose deaths among Black New Yorkers from 15.7 per 100,000 in 2019 to 50.7 per 100,000 in 2022. Overdose mortality among Latino New Yorkers more than doubled from 21.4 to 44.7 per 100,000 during the same time [5]. The majority of these overdose fatalities involved the synthetic opioid fentanyl (which is often illicitly manufactured and can be hundreds of times more powerful than morphine [6]), as well as other adulterants including xylazine [7] and benzodiazepines [8]. All can be present in a drug without people’s knowledge.

Adulterants can be added to illicit drug supplies as “bulking agents” designed to inexpensively increase the quantity of a substance before sale or to enhance the psychoactive effects of a drug [9]. For example, fentanyl is often added to heroin or other opioid products to maximize potency at a lower cost, but because the effects of fentanyl are short-lasting, other adulterants (ie, xylazine or benzodiazepines) may be added to prolong fentanyl’s effects, giving the drug more “legs” [10]. Xylazine use can result in rapid loss of consciousness and painful skin ulcers [9], along with blackouts that leave people with no memory for extended periods of time [11]. Benzodiazepines may cause heightened drug dependence and are associated with severe physiological and psychological withdrawal symptoms [8] that last longer than opioid withdrawal. Fentanyl is increasingly found in stimulants, including cocaine and methamphetamine, and in illicitly manufactured pharmaceutical pills (eg, counterfeit oxycodone or Xanax) [12]. Adulterated substances can have unpredictable effects, especially when individuals consume multiple adulterants and are, therefore, exposed to a combination of drugs they did not intend to consume [12,13]. Fentanyl was present in 81% of New York City overdose deaths in 2022, and cocaine was present in 53% [5]. In the same year, 47% of New York City overdose deaths involved a combination of opioids and cocaine and 19% involved both opioids and xylazine [5].

According to a Centers for Disease Control and Prevention (CDC) study of fatal overdose in the United States, the monthly percentage of fentanyl-involved deaths with xylazine detected increased by 276% from January 2019 through June 2022 [14]. In a separate study of fatal overdose events in 38 states and the District of Columbia from January through June of 2020, more than 92% of benzodiazepine deaths also involved fentanyl [15]. Xylazine deaths have become common in Philadelphia [3] and are increasing in New York City [15] and nationwide. The White House has designated fentanyl combined with xylazine an “Emerging Threat to the United States” [16].

Local health departments and other agencies that serve people who use drugs have made significant efforts to saturate communities with naloxone to reverse overdose events and fentanyl test strips (FTS) so that people can know what adulterants may be present in a batch of drugs prior to consumption. Existing research has shown that among people who use drugs, FTS use is both feasible and acceptable [17-19] and, that in some cases, people desired xylazine test strips (XTS) [11].

A recent survey of North American drug-checking services shows that 16 organizations have served more than 125,000 people and checked almost 50,000 drug samples since 2014 [20]. This includes the use or distribution of FTS and XTS, which indicate the presence of fentanyl or xylazine, respectively, as well as Fourier-transform infrared spectroscopy (FTIR), which can identify all the chemicals in a substance in proportion to the total sample (eg, what percentage of a checked sample is fentanyl and what percentage is xylazine). FTS and XTS are relatively inexpensive (a package of 10 XTS can be purchased via Amazon.com for US $15.99 [21]; similar products are often given away free of charge by agencies that provide services to people who use drugs) and can be easily distributed for use at home or in other off-site settings. FTIR devices are more expensive and require a trained technician to operate and interpret results. Syringe service programs (SSPs) or other community outreach settings often provide FTIR access in fixed locations such as drop-in centers [20]. Both the New York State Office of Substance Use Services and The New York City Department of Health and Mental Hygiene distribute no-cost FTS and XTS along with detailed instructions on how to use them [22,23] and partner with community-based organizations to provide no-cost FTIR services.

However, many of those who could benefit most from drug checking may not be aware of the risks associated with adulterants or know how to access necessary supplies or service providers. Others may have heard about adulterants but discount their own potential risk. In some areas, adulterants have become so prevalent that people who intentionally use fentanyl now face risks from xylazine or other adulterants that may be mixed into their drug purchases, underscoring the need for and potential value of routine drug checking [13].

In addition, many people who use drugs may be well aware of the presence of adulterants in the illicit drug supply and the dangers they cause but elect not to use drug-checking technologies. This can include unhoused people who believe that checking their drugs in public spaces before use would invite unwanted scrutiny from police or others and reinforce public stigma. There are also people with extensive drug use experience who believe they can identify adulterated drugs by color, texture, or taste and thus view drug checking as something unnecessarily burdensome rather than a lifesaving harm reduction intervention [24].

To better understand people who use drugs’ knowledge of adulterants in the drug supply, as well as barriers to and facilitators of drug checking, our team conducted a series of in-depth qualitative interviews with people who use drugs in New York City to learn about their experiences as part of this formative and exploratory study. Our ultimate goal is to develop technology-based interventions designed to increase drug checking and other overdose prevention practices to mitigate harm from the toxic unregulated drug supply. In the past, our team has created intervention content delivered via tablet computers and text messages to encourage positive health behaviors, including increased testing for HIV and hepatitis C [25,26], uptake of take-home naloxone kits [27], and vaccination against COVID-19 among people who inject drugs [28,29]. We now seek to use a similar approach, guided by accepted models of behavior change and empirically derived theories of multimedia learning [30], to encourage the use of drug-checking services. In keeping with the community-based Participatory Education and Research into Lived Experience (PEARLE) intervention development methodology [31], we first set out to identify barriers to drug-checking in order to then begin the process of designing interventions to encourage routine drug-checking.

## Methods

### Overview

Two interviewers, who had prior research experience working with people who use drugs, conducted a series of individual, semistructured interviews with a convenience sample of people who reported the use of illicit substances within the past 90 days. During July and August of 2023, participants were recruited in areas of parks where people who use drugs are known to congregate, and where there is high drug activity. Staff members initially approached people in these areas and explained that the team was from New York University and interested in learning about people’s knowledge of the drug supply, their use of harm reduction measures, and barriers to uptake of drug checking; they then asked if they would be willing to participate in a confidential interview about their experiences. Before each interview, potential participants completed a paper-based substance use screening based on the World Health Organization (WHO) Alcohol, Smoking and Substance Involvement Screening Test (ASSIST) [32]. The screening listed different substances and asked how often the participant had used them in the past 3 months. Potential participants read through the list independently and circled their answers on the sheet. People were eligible to participate if they self-reported using at least 1 of the following: cocaine, amphetamine-type substances, inhalants, sedatives, benzodiazepines, hallucinogens, prescription opioids other than as prescribed by a doctor, or heroin.

### Ethical Considerations

Staff obtained verbal informed consent prior to study participation. All interviews were audio recorded in quiet areas of a park for later analysis and lasted for approximately 30 minutes. No identifying details were collected or recorded by our study team; all data are anonymous. Participants were given US $20 cash as compensation for their time. All protocols, consent documents, and the interview guide were reviewed and approved by the Biomedical Research Alliance of New York institutional review board (submission #215627).

### Interview Guide

The interview guide was developed by members of the study team, including members who have experience working with people who use drugs and are involved in FTS and XTS distribution throughout New York City. The guide contained sections examining participants’ knowledge of fentanyl, xylazine, and benzodiazepines. Drug types were referred to by pharmacological terms and informal names, for example, xylazine was described as “tranq” and xylazine mixed with heroin was described as “tranq dope.” Benzodiazepines were additionally referred to as “benzos” and by product brand names, such as Xanax and Klonopin.

The interview guide also contained questions about participants’ knowledge of, and experience with, drug testing strips and spectrometers, as well as whether they had ever received services at an SSP or overdose prevention center. The guide also contained questions about whether participants had used drugs that had been adulterated with fentanyl, xylazine, and benzodiazepines, and how they knew their drugs did or did not contain adulterants. Interview guide questions also examined what participants would do if they learned that the drugs they purchased contained adulterants.

### Coding and Analysis

All interviews were transcribed and then uploaded into the MAXQDA software (VERBI Software) platform for coding and analysis. Transcripts were analyzed by thematic analysis. Two of the authors who also conducted the interviews coded and analyzed the transcripts and met weekly with the larger team to discuss codes and emerging interpretations. The initial codebook consisted of a priori constructs (based on the aims of the study and the interview guide) and emerging themes (that were related to the study aims but not specifically anticipated). The 2 authors each read the same 3 transcripts and developed a preliminary code guide that included items and domains from the study aims. At weekly meetings, the larger team (coauthors) and the 2 coders discussed and refined the code list and checked for consistency of interpretations and reconciled any discrepancies. This process was repeated for an additional round of coding with 3 new transcripts resulting in the penultimate codebook. The remainder of the transcripts were coded by the 2 interviewers with the team meeting to discuss discrepancies and then finalize the code list [33].

In total, 22 interviews were conducted with participants ranging in age from 21 to 66 years. Participants self-identified as Hispanic or Latino (n=7), including 2 who identified as White Hispanic or Latino, 4 who identified as Black or African American Hispanic or Latino, and 1 who identified as multiracial (American Indian/Alaska Native and Native Hawaiian or Other Pacific Islander). For those who identified as non-Hispanic or Latino (n=12), 3 identified as White, 6 identified as Black or African American, 1 identified as American Indian/Alaska Native, 1 identified as multiracial (White and American Indian/Alaska Native), and 1 wrote “Jamaican/Native American” for race. Three participants declined to report their ethnicity. In terms of gender, 12 self-identified as male, 3 as female, and 7 declined to state.

## Results

### Overview

The 22 participants reported an average of 20.7 (SD 13) years of using drugs. Almost three-quarters (n=16, 73%) of participants reported using both opioids (heroin and prescription opioids other than as prescribed by a doctor) and stimulants (cocaine and amphetamine-type substances). Cocaine use in the past 90 days was reported by approximately 82% (n=18) of participants and heroin by 55% (n=12). More than one-third (n=8, 36%) reported using fentanyl on purpose in the past 90 days.

### Knowledge of Adulterants in the Drugs Supply

Despite the resources allocated toward overdose prevention efforts in New York, a city with a robust harm reduction infrastructure, interviews with participants suggest there are considerable knowledge gaps about the local drug supply, along with significant misinformation, uneven uptake of overdose prevention and drug-checking resources (eg, naloxone and FTS), and other barriers that may hinder overdose response efforts in the city.

Most participants reported they were unfamiliar with xylazine and benzodiazepines. Few were able to describe the effects of either drug type. One participant, a 58-year-old, Black Hispanic, male individual with 40 years of drug use experience, described trying xylazine once and quickly losing consciousness. He said he would never buy drugs again from the people who sold him xylazine. Another participant, a 44-year-old, non-Hispanic, Black, female individual using drugs for 20 years, reported seeing news segments about xylazine on television and reading about it in a newspaper: “there was someone that had tried it and the next day they had a hole in their leg.” A third participant appeared to conflate xylazine with K2 and thought it made people combative and gave them superhuman strength.

While almost all participants reported they had heard of fentanyl, most could not identify it as an opioid or describe the effects it would have on a person other than saying “that it’s bad” or it “kills people.” One participant, a 56-year-old, Black, non-Hispanic interviewee who reported 20 years of drug use and did not specify their gender, identified fentanyl as a substance used to “stretch drugs to make them bigger” in order to “make more money off of ‘em.” A 57-year-old, male, non-Hispanic, multiracial participant who reported 35 years of drug use reported purposely seeking out fentanyl twice, so he could “see what the big fuss was about.”

Many participants did not appear to understand that fentanyl, or other adulterants, could be present in drugs they used without their knowledge. One participant who reported 10 years of drug use expressed a combination of surprise and disbelief when an interviewer questioned whether they or people close to them, could have consumed adulterated substances without knowing.

Interviewer: And so, have you ever used Xylazine?

Interviewee: Not at all. I wouldn’t be talking to you right now. I probably wouldn’t be the same.

Interviewer: How do you know that you haven’t?

Interviewee: I don’t know. [Your questions] spooked me on that. I don’t even know how to answer that question...that question really got me thinking...

Interviewer: We’ll talk about a couple of ways...

Interviewee: So, wait, hold on. So, there’s a chance that...we could have took these drugs and didn’t know, we just have been getting lucky over the years?

28-year-old, Black, Hispanic participant; gender not specified

### Drug-Checking Experience

Some interviewees reported regularly checking their drugs for adulterants (1 participant wanted to purchase fentanyl and used testing strips to confirm fentanyl presence; another described an “ex-fiancé” who sold drugs and “would offer people test strips so that they could test his supply so that they know that his stuff is good”). However, the majority of people we interviewed reported not checking their drugs for adulterants before use. Interviews show many participants were aware of drug testing strips, but that FTS and XTS were not consistently used, largely for reasons of time and convenience.

The 57-year-old, male, non-Hispanic, multiracial participant who reported 35 years of drug use explained that he knew drug testing strips were available, but “when you want to get high, you don’t have time for that.” The same participant reported that issues of convenient supply access became particularly acute when using drugs in public, which is especially problematic for people who are unhoused.

If I was in the park and I want to go get a package of powdered cocaine, and I’m going to come back in the park and hang out and get high and enjoy the day, if I knew that my friend over there had [the] strip them on them. “Yo, yo, let me get one of your strips” I will test it, and then I will go about my day. But that’s not readily available like cigarettes and weed and rolling papers. So, if that was more accessible, I think maybe it would be a lot helpful, more helpful.

Interviews also underscore that while some participants knew adulterants were increasingly present, and could cause serious health risks including death, they still viewed drug checking as something other than an established norm. One participant who reported 9 years of drug use said that even though a friend had died from using cocaine adulterated with fentanyl, they were completely unaware of people in their social circles testing drugs before use.

I haven’t heard nobody that I associate with using the test strips at all. That’s like something new honestly because I haven’t heard nobody using test strips. People still dying from fentanyl. People still getting fake Percocets and fake stuff.

25-year-old, multiracial, non-Hispanic participant; gender not specified

### Not Recognizing the Need for Drug Checking

The perceived safety of cocaine as a nonopioid emerged as another common reason for not checking drugs. Some participants were adamant that despite years of using drugs, they were personally not at risk from fentanyl because they exclusively smoked crack or were not “an addict.” One 56-year-old, Black, male participant (ethnicity not specified) reported 38 years of drug use and said he would stop “if they put it in cocaine. But as far as I know they don’t put in cocaine, they put [in] heroin.”

Similarly, the 56-year-old, Black, non-Hispanic interviewee who reported 20 years of drug use and did not specify gender, said they had no need to test for adulterants:

because I don’t use those type of drugs anyhow. No heroin, I smoke a little crack cocaine and marijuana a little. I’m not physically addicted to nothing. I’m not really mentally addicted to nothing.

Similar to the previous quote, other participants perceived crack cocaine as especially safe from adulterant-related overdose risk.

With your crack, if you smoke crack, usually people go to person that they’ve been messing with, that’s really reliable, that’s usually known for having a good product, that’s loyal to they customer, you know, certain things like that, you know, just morals and certain things. That just helps. It just makes it a lot more safer. A lot more safer.

25-year-old, multiracial, non-Hispanic participant; gender not specified

The theme of always purchasing from a single, highly trusted source emerged as an especially strong barrier to drug checking. Multiple participants described close relationships with a drug seller who they relied on to keep them safe by selling quality, unadulterated substances. One person even referred to the drug sellers he frequents as his “people” and his “family.”

I mean, close ones, loved ones, you know, what I’m saying? Those are the people that I deal with for my drug use as far as purchasing my drugs and stuff like that. So when I’m dealing with my family and stuff like that, close ones, I trust them and I know that they don’t [put] fentanyl in it.

42-year-old, Black, non-Hispanic, male participant with 12 years of reported drug use

Among the smaller number of participants who reported regularly checking drugs for adulterants, many cited positive interactions with health care providers or harm reduction outreach teams. Of those participants who reported checking drugs prior to consumption, a substantial number described receiving services from an SSP, either on-site or in an outreach setting. Some were able to name multiple harm reduction agencies and their locations. One described using a drug testing device (possibly an FTIR or mass spectrometer [34]) that displayed the percentage of different chemicals in a sample of drugs they brought to an SSP. Other participants described outreach teams coming to parks where they spend their time and noted the benefits of relationships that harm reduction outreach teams can develop with people who use drugs.

They come out here maybe about four times a week, you know, they mainly got everything that we need to make sure...we do things properly...make sure that we inject safe...you know, test our shit. So you know, this way we know what we put in our bodies...Honestly, I forget the names of the programs because, you know, that’s the type of thing doesn’t really matter to me. It all matters that they’re there for me and they’re helping.

28-year-old, White, non-Hispanic, female participant with 9 years of reported drug use

### If Drugs Were Found to Contain Adulterants

Like the earlier participant who said he would never buy drugs again from the person who sold him xylazine, when presented with a similar hypothetical situation, participants frequently said they would not purchase drugs from someone who sold adulterated substances, and they would not purchase drugs they knew contained adulterants. Two participants said they would respond with physical violence if someone sold them adulterated drugs (eg, “If they don’t give you what you pay for, then you have a right to go after them”).

One 51-year-old participant who identified as male, non-Hispanic, American Indian/Alaska Native, and reported 13 years of drug use experience said he would take drugs back to the seller if they tested positive for adulterants because “drug dealers should know what they put into their drugs.” Only 1 participant, a 25-year-old, non-Hispanic male individual, said he would dispose of drugs if he learned they contained adulterants.

## Discussion

### Principal Findings

Our interviews with people who use drugs in New York City indicate a lack of knowledge of adulterants in the local drug supply, complicated by widespread misinformation about the risks of opioid overdose. Although these interviews show that a smaller number of participants regularly check drugs for adulterants, most do not. Interview data also underscore the value of outreach teams and other care providers who encourage the use of harm reduction techniques including drug checking.

As detailed earlier, 1 participant reported that no one they associate with uses drug testing strips at all. Moreover, multiple participants expressed skepticism and surprise that fentanyl, xylazine, or benzodiazepines might already be present in the drugs they use. Given the increasing prevalence of highly dangerous adulterants in our nation’s drug supply this creates obvious, and all too often fatal, health risks for people who use drugs.

The finding that most participants interviewed (16/22, 73%) report currently using both opioids and stimulants further highlights the importance of drug checking prior to consumption, as well as the complexity of efforts to increase consistent checking among the different populations who are now at risk. For example, increased rates of fentanyl overdose among people who use cocaine and other stimulants, including people without a history of intentional opioid use [35] who have not built up a physical tolerance to opioids [36], indicate the escalating danger faced by people who use drugs but may not understand how the drug supply has changed in recent years. Prior to fentanyl, opioid overdose was less of a concern for people who use stimulants. As a result, many people who use cocaine, such as the participant who reported 38 years of drug use and was adamant people do not put fentanyl in cocaine, remain unaware of their current risk and could especially benefit from drug-checking services [36].

Likewise, people who use pills obtained without a prescription may not know that counterfeit pharmaceuticals frequently contain potentially deadly amounts of fentanyl and benzodiazepines [12]. Thus, there is an immediate need for educational outreach that emphasizes the need for multiple harm reduction techniques (eg, consistently carrying naloxone to reverse overdose events) [35] and is tailored to different populations, including people who use drugs and were previously not at risk for opioid overdose, and especially people who use drugs and do not currently receive harm reduction services [12].

Further, nonstigmatizing education is needed to ensure those at risk not only understand the dangers of an adulterated supply but act upon these risks to protect their health [11]. As described in the quote above, many people who use drugs are well aware of deaths due to fentanyl and other adulterants in heroin and counterfeit pills, yet still do not check their drugs. In some cases, this is due to a lack of convenient access to drug-checking supplies or a place to test drugs out of public view. In other cases, people do not check their drugs because they view it as a waste of time or product [24,37] or because they fear it could expose them to legal consequences if they are found in possession of a controlled substance or test strips [13]. Indeed, in some states, drug-checking strips are considered drug paraphernalia and are illegal (eg, Iowa, Indiana, and North Dakota) [38]. In other words, the real and perceived benefits of drug checking must outweigh the risks of accessing services [39] or they will remain underused.

Thus, changing drug use behavior to encourage routine drug checking prior to consumption requires not only reaching the most vulnerable, which in itself presents a significant challenge, but delivering theory-guided intervention content that people find worthy of their attention and credible enough to act upon [40,41]. It will also require ensuring that people who use drugs can easily access the drug-checking resources they need, when they need them, given the context and constraints within which they are operating. This includes addressing structural vulnerabilities faced by people who use drugs, such as poverty (people might still use drugs found to be adulterated because they cannot afford to replace them) and the need to consume drugs to avoid painful withdrawal symptoms [37]. Related findings described above may help explain why only 1 person interviewed for the study said they would discard drugs shown to contain adulterants, and only 2 people said they would confront a dealer who sold them adulterated drugs, even though multiple participants spoke in detail about the dangers of consuming adulterated substances. Existing research has shown that poverty and a lack of consumer protections in an unregulated drug market greatly limit options for recourse—to put it mildly, people cannot simply return a defective purchase without any consequences [37].

At the same time, interviews reinforce the importance of trusted relationships many people who use drugs have developed with the people they buy drugs from. Multiple study participants expressed great confidence that drug sellers they frequent would not add fentanyl or other adulterants to their product. This potential “over trusting” [42] creates clear risks—if people do not test their drugs, it may be impossible to know which adulterants they are consuming or in what quantity (eg, how much of what they are taking is actually heroin and how much is fentanyl, xylazine, or something else). A recent study in New York City of more than 300 people who inject drugs found that while only 18% reported intentional fentanyl use, a urine toxicology screening showed 83% tested positive for fentanyl [43].

### Limitations

The primary limitations of the study are the relatively small sample size and the fact that all participants were recruited from 2 public parks in New York City. However, qualitative interviews for the study were not meant to be generalizable beyond our specific sample. Nonetheless, our findings highlight the need for expanded outreach and education [44] and may contribute to the development of more effective interventions to encourage people to check their drugs for adulterants prior to use.

### Conclusions

The finding that participants who reported regularly checking drugs also described positive relationships with harm reduction workers and outreach teams is especially encouraging. Among the smaller number of people who were knowledgeable about and reported the use of drug-checking supplies, the most common source of knowledge and access was some type of harm reduction organization or outreach effort. Increased outreach may be especially important to reach people who regularly use drugs yet are not affiliated with an SSP or other type of care provider. In particular, outreach may prove especially valuable for people who consume drugs via noninjection methods [12] and may believe an SSP is of no use to them (people who sniff drugs or swallow pills also face clearly increased overdose risk due to adulterants and may benefit from drug checking and other harm reduction services offered at SSP locations).

The finding that a drug seller provided test strips so his customers could independently verify he sold “good” drugs aligns with prior research [37,45], and suggests that building upon established relationships between people who use drugs and people who sell drugs may be a good way to strengthen and extend harm reduction efforts. Accordingly, future research can examine how technology-based intervention content developed with extensive input from community members and delivered by trusted individuals (eg, outreach workers, other people who use drugs, and people who sell drugs) may potentially increase drug checking and additional behaviors that help people protect themselves and others against overdose [46]. Once developed, further research is warranted to examine how this intervention content can be most effectively disseminated, along with drug-checking supplies, to high-need populations that may not currently be reached by existing overdose prevention efforts.

## Abbreviations

ASSIST: Alcohol, Smoking and Substance Involvement Screening Test
CDC: Centers for Disease Control and Prevention
FTIR: Fourier-transform infrared spectroscopy
FTS: fentanyl test strip
MMS: multimedia messaging service
PEARLE: Participatory Education and Research into Lived Experience
SSP: syringe service program
WHO: World Health Organization
XTS: xylazine test strip

## Data Availability

The data sets generated and analyzed during this study are not publicly available in order to protect participant privacy but are available from the corresponding author on reasonable request.

## References

[1] Hedegaard H, Miniño AM, Spencer MR, Warner M (2021). Drug overdose deaths in the United States, 1999-2020. NCHS Data Brief.

[2] Zagorski CM, Hosey RA, Moraff C, Ferguson A, Figgatt M, Aronowitz S, Stahl NE, Hill LG, McElligott Z, Dasgupta N (2023). Reducing the harms of xylazine: clinical approaches, research deficits, and public health context. Harm Reduct J.

[3] CDC Provisional drug overdose death counts. Vital Statistics Rapid Release 2022.

[4] Askari MS, Bauman M, Ko C, Tuazon E, Mantha S, Harocopos A, Hygiene NYCDoHaM (2021). Unintentional Drug Poisoning (Overdose) Deaths.

[5] Health N (2022). Unintentional drug poisoning (overdose) deaths. NYC Epi Data Brief.

[6] O'Donnell J, Gladden RM, Mattson CL, Hunter CT, Davis NL (2020). Vital signs: characteristics of drug overdose deaths involving opioids and stimulants - 24 states and the District of Columbia, January-June 2019. MMWR Morb Mortal Wkly Rep.

[7] Quijano T, Crowell J, Eggert K, Clark K, Alexander M, Grau L, Heimer R (2023). Xylazine in the drug supply: emerging threats and lessons learned in areas with high levels of adulteration. Int J Drug Policy.

[8] Russell C, Law J, Bonn M, Rehm J, Ali F (2023). The increase in benzodiazepine-laced drugs and related risks in Canada: the urgent need for effective and sustainable solutions. Int J Drug Policy.

[9] Holt AC, Schwope DM, Le K, Schrecker JP, Heltsley R (2023). Widespread distribution of xylazine detected throughout the United States in healthcare patient samples. J Addict Med.

[10] (2023). Xylazine: what clinicians need to know. Department of Health.

[11] Reed MK, Imperato NS, Bowles JM, Salcedo VJ, Guth A, Rising KL (2022). Perspectives of people in Philadelphia who use fentanyl/heroin adulterated with the animal tranquilizer xylazine; making a case for xylazine test strips. Drug Alcohol Depend Rep.

[12] O'Donnell Julie, Tanz LJ, Miller KD, Dinwiddie AT, Wolff J, Mital S, Obiekwe R, Mattson CL (2023). Drug overdose deaths with evidence of counterfeit pill use - United States, July 2019-December 2021. MMWR Morb Mortal Wkly Rep.

[13] Grace Rose C, Pickard AS, Kulbokas V, Hoferka S, Friedman K, Epstein J, Lee TA (2023). A qualitative assessment of key considerations for drug checking service implementation. Harm Reduct J.

[14] Kariisa M, O'Donnell J, Kumar S, Mattson CL, Goldberger BA (2023). Illicitly manufactured fentanyl-involved overdose deaths with detected xylazine - United States, January 2019-June 2022. MMWR Morb Mortal Wkly Rep.

[15] Liu S, O'Donnell J, Gladden RM, McGlone L, Chowdhury F (2021). Trends in nonfatal and fatal overdoses involving benzodiazepines - 38 States and the District of Columbia, 2019-2020. MMWR Morb Mortal Wkly Rep.

[16] (2023). Biden-Harris administration designates fentanyl combined with xylazine as an emerging threat to the United States. ONDCP.

[17] Reed MK, Roth AM, Tabb LP, Groves AK, Lankenau SE (2021). "I probably got a minute": perceptions of fentanyl test strip use among people who use stimulants. Int J Drug Policy.

[18] Krieger MS, Yedinak JL, Buxton JA, Lysyshyn M, Bernstein E, Rich JD, Green TC, Hadland SE, Marshall BDL (2018). High willingness to use rapid fentanyl test strips among young adults who use drugs. Harm Reduct J.

[19] Sherman SG, Morales KB, Park JN, McKenzie M, Marshall BD, Green TC (2019). Acceptability of implementing community-based drug checking services for people who use drugs in three United States cities: Baltimore, Boston and Providence. Int J Drug Policy.

[20] Park JN, Tardif J, Thompson E, Rosen JG, Lira JAS, Green TC (2023). A survey of North American drug checking services operating in 2022. Int J Drug Policy.

[21] Rapid response xylazine test strips - pack of 10 test strips - test liquids and powders for presence of xylazine. Brand: BTNX Inc.

[22] Fentanyl. NYCDOHMH.

[23] How to test your drugs using xylazine test strips. NYCDOHMH.

[24] Aronson ID, Bennett AS, Ardouin-Guerrier M, Baus JE, Stern LS, argas-Estrella B, Gibson B (2023). Known fentanyl use and potential reasons for non-use of fentanyl test strips among people who use drugs. Annual Meeting of the College of Problems on Drug Dependence.

[25] Aronson ID, Marsch LA, Rajan S, Koken J, Bania TC (2015). Computer-based video to increase HIV testing among emergency department patients who decline. AIDS Behav.

[26] Aronson ID, Bennett A, Marsch LA, Bania TC (2017). Mobile technology to increase HIV/HCV testing and overdose prevention/response among people who inject drugs. Front Public Health.

[27] Aronson ID, Freeman R, Des Jarlais D, Bennett AS (2020). Video to increase naloxone uptake and use among PWUO who decline.

[28] Aronson ID, Bennett AS, Giles SA, Ardouin-Guerrier M, Baus JEB, Gibson BV (2023). Development of text message-based intervention content for people who inject drugs: opportunities and challenges.

[29] Aronson ID, Bennett AS, Ardouin-Guerrier M, Rivera-Castellar G, Gibson B, Santoscoy S, Vargas-Estrella B (2022). How vaccine ambivalence can lead people who inject drugs to decline COVID-19 vaccination and ways this can be addressed: qualitative study. JMIR Form Res.

[30] Aronson ID, Marsch LA, Acosta MC (2013). Using findings in multimedia learning to inform technology-based behavioral health interventions. Transl Behav Med.

[31] Aronson ID, Bennett AS, Ardouin-Guerrier M, Rivera-Castellar GJ, Gibson BE, Vargas-Estrella B (2022). Using the participatory education and research into lived experience (PEARLE) methodology to localize content and target specific populations. Front Digit Health.

[32] The ASSIST project - Alcohol, Smoking and Substance Involvement Screening Test. World Health Organization.

[33] Miles MB, Huberman AM (1994). Qualitative Data Analysis: An expanded Sourcebook (2nd Ed.).

[34] Ti L, Tobias S, Lysyshyn M, Laing R, Nosova E, Choi J, Arredondo J, McCrae K, Tupper K, Wood E (2020). Detecting fentanyl using point-of-care drug checking technologies: a validation study. Drug Alcohol Depend.

[35] Tilhou AS, Zaborek J, Baltes A, Salisbury-Afshar E, Malicki J, Brown R (2023). Differences in drug use behaviors that impact overdose risk among individuals who do and do not use fentanyl test strips for drug checking. Harm Reduct J.

[36] Tobias S, Ferguson M, Palis H, Burmeister C, McDougall J, Liu L, Graham B, Ti L, Buxton JA (2024). Motivators of and barriers to drug checking engagement in British Columbia, Canada: findings from a cross-sectional study. Int J Drug Policy.

[37] Bardwell G, Boyd J, Tupper KW, Kerr T (2019). "We don't got that kind of time, man. we're trying to get high!": exploring potential use of drug checking technologies among structurally vulnerable people who use drugs. Int J Drug Policy.

[38] Legality of drug checking equipment in the United States. The Network for Public Health Law.

[39] Gozdzialski L, Wallace B, Hore D (2023). Point-of-care community drug checking technologies: an insider look at the scientific principles and practical considerations. Harm Reduct J.

[40] Bandura A, DiClemente JPR (1994). Social cognitive theory and exercise of control over HIV infection. Preventing AIDS Theories and Methods of Behavioral Interventions.

[41] Fisher JD, Fisher WA, DiClemente JPR (2000). Theoretical Approaches to Individual-Level Change in HIV Risk Behavior, in Handbook of HIV Prevention.

[42] Swartz JA, Lieberman M, Jimenez AD, Mackesy-Amiti ME, Whitehead HD, Hayes KL, Taylor L, Prete E (2023). Current attitudes toward drug checking services and a comparison of expected with actual drugs present in street drug samples collected from opioid users. Harm Reduct J.

[43] McKnight C, Weng CA, Reynoso M, Kimball S, Thompson LM, Jarlais DD (2023). Understanding intentionality of fentanyl use and drug overdose risk: findings from a mixed methods study of people who inject drugs in New York City. Int J Drug Policy.

[44] Tanz LJ, Gladden RM, Dinwiddie AT, Miller KD, Broz D, Spector E, O'Donnell J (2024). Routes of drug use among drug overdose deaths - United States, 2020-2022. MMWR Morb Mortal Wkly Rep.

[45] Bardwell G, Boyd J, Arredondo J, McNeil R, Kerr T (2019). Trusting the source: the potential role of drug dealers in reducing drug-related harms via drug checking. Drug Alcohol Depend.

[46] Bennett AS, Scheidell J, Bowles JM, Khan M, Roth A, Hoff L, Marini C, Elliott L (2022). Naloxone protection, social support, network characteristics, and overdose experiences among a cohort of people who use illicit opioids in New York City. Harm Reduct J.

